# Impact of a diagnostic therapeutic educational pathway program for asthma management in preschool children

**DOI:** 10.1186/s13052-021-00992-y

**Published:** 2021-03-10

**Authors:** Sebastiano Guarnaccia, Cristina Quecchia, Andrea Festa, Michele Magoni, Giuseppe Zenoni, Emanuele D’Agata, Valentina Brivio, Elena Zanardini, Carmelo Scarcella, Valeria Gretter, Susanna Facchetti, Cinzia Gasparotti, Ada Pluda, Malica Frassine, Rosa Maria Limina, Raffaele Spiazzi, Raffaele Badolato, Bruce Bender, Francesco Donato

**Affiliations:** 1 Department of Clinical and Experimental Sciences, University of Brescia, Brescia, Italy; 2 Centro “Io e l’Asma”, Ospedale dei Bambini, ASST Spedali Civili, Brescia, Italy; 3 Department of Medical and Surgical Specialities, Radiological Sciences and Public Health, University of Brescia, University of Brescia, Brescia, Italy; 4 Brescia Health Protection Agency, Brescia, Italy; 5 Post-Graduate School of Public Health, University of Brescia, Brescia, Italy; 6 ASST Garda, Desenzano, Italy; 7 ASST Lariana, Como, Italy; 8 I.R.C.C.S. Giannina Gaslini, Genoa, Italy; 9 Department of Pediatrics, Center for Health Promotion National Jewish Health, Colorado Denver, USA

**Keywords:** Infant, Children, Wheezing, Preschool asthma, Diagnostic-therapeutic-educational pathway, GINA guidelines, Patient and family education, Before-after evaluation

## Abstract

**Background:**

Preschool children with clinically-diagnosed asthma have a higher rate of emergency department visits and consume more resources for management than older children. However, no clinical trials have yet been performed measuring the impact of a combined diagnostic, therapeutic and educational pathway regimen for evaluation of wheezing control in children aged less than 6 years.

The purpose of the present study was to assess the impact of a pediatric program developed in Italy, the Diagnostic Therapeutic Educational Pathway (DTEP), for asthma management in children less than 6 years old attending an asthma referral center.

**Methods:**

This is a retrospective population-based cohort study performed in children with asthma aged 0–5 years, attending at “Io e l’Asma center”, Brescia, Italy between September 2007 and December 2014. The incidence rates (IRs) of hospitalization, emergency room visits, use of outpatient services and drug usage for dyspnea, wheezing, or respiratory symptoms were evaluated for time periods prior to and after DTEP intervention.

**Results:**

A total of 741 patients, aged 0–5 years completed the DTEP, including 391 and 350 children aged 0–2 and 3–5 years, respectively. The percentage of children aged 0–2 and 3–5 years showing improved control of wheezing symptoms during the 1st to 3rd visit interval as a result of the DTEP intervention increased from 39.5 to 60.9% and from 25.5 to 75.5%, respectively. During these periods, the IRs showed a significant decrease for all outcomes, from-8.6% to − 80.4%. Although specific IRs for drug prescriptions declined, particularly for LABA plus corticosteroids, antibiotics, and systemic corticosteroids, they increased for SABA, inhaled corticosteroid and leukotriene receptor antagonist usage.

**Conclusions:**

The results suggest that a real-world assessment of the integrated DTEP program for preschool children provides evidence for improved wheezing control and reduction of adverse therapeutic related outcomes.

## Background

Recurrent wheezing in children aged 5 years or younger is one of the most common chronic childhood symptoms and the main reason for consumption of pediatric health resources in Western countries, as measured by emergency department visits and hospitalizations [1]. Asthma diagnosis is classically based on clinical history, symptoms and treatment responses. However, establishing a diagnosis of asthma in children less than 6 years of age may present a challenge since respiratory symptoms, including wheezing or cough, are also commonly seen in children without asthma, particularly in those younger less than 2 years [2, 3]. In fact, of the 30–50% of preschool children who experience episodes of wheezing, fewer than half have childhood asthma [4]. The Canadian Thoracic Society and Canadian Pediatric Society established clinical criteria for diagnosis of asthma in children one to 5 years of age with frequent asthma-like symptoms or recurrent episodes with asthma-like signs [5, 6].

Some studies showed that preschool children with clinically-diagnosed asthma not only have higher rates of emergency department visits and hospitalization but also consume more resources for asthma management than those aged 6 years or older [7–9]. Furthermore, since children aged less than 6 years with wheezing illnesses are at high risk of impaired lung growth and pulmonary function in childhood and adulthood [5], early diagnosis and treatment of wheezing is essential in preschool children mild persistent asthma of recent onset for reducing morbidity by decreasing the risk of severe exacerbations and improving control [10].

In a series of previous publications, we described the successful diagnostic and therapeutic outcomes of patient management of adolescent and teen-age patients with asthma using a pediatric self-management program that we developed in Italy referred to as the Diagnostic Therapeutic Educational Pathway (DTEP) [11–15]. The purpose of the present study was to evaluate the effectiveness of the DTEP in a cohort of younger preschool children with wheezing by comparing the frequency of wheezing-related outcomes before and after intervention with this patient self-management program.

## Methods

### Patients

The Center “Io e l’Asma” is an outpatient pediatric asthma center at the University of Brescia, which provides a Diagnostic Therapeutic Educational Pathway (DTEP), designed to obtain and maintain control of wheezing by focusing on child and family autonomy and relying on a strong collaboration between specialists and primary care physicians, where we organized several meetings to share the DTEP following the GINA guidelines, during the last 15 years [11–15]. The DTEP consists of three specialist evaluations at the Center, with an 8–12 weeks’ interval between them, and two follow-up visits at 6 and 12 months after the last evaluation. After each specialist’s evaluation, the child is referred to his/her primary care physician for documenting the symptoms control by clinical evaluation and adjusting therapy for 6–8 weeks based upon the degree of wheezing and respiratory compromise. After the first visit, the patients and their parents receive an educational and therapeutic educational assessment in a dedicated and structured setting (30 min) by a health care assistant. Preventive measures, early recognition of symptoms, an action plan for managing attacks, use of drugs and devices, maintaining a healthy lifestyle and keeping a diary for monitoring symptoms are included in the discussion. At subsequent visits, the specialist evaluates the levels of symptom control according to the GINA preschool children guidelines [1]: daytime and night waking, use of reliever drugs, and activity limitation. Treatment adherence is also evaluated, and modification of daily pharmacologic therapy is performed as needed.

### Study design

This was a retrospective cohort study in which all children attending the Center received the DTEP intervention according to the following criteria: a) age between 0 and 5 years; b) at least one visit to the Center “Io e l’Asma” between 1st September 2007 and 31st December 2014; and c) residence in Brescia province, Lombardy region, Italy. Demographic (patients’ code, social security numbers, date of birth and place of residence) and clinical data (physical examinations, diagnostic tests, therapies and asthma exacerbations) were collected from the Center database. The study was approved by the Province Ethics Committee at “ASST Spedali Civili di Brescia”, Brescia, Italy (registration number 2046) on 15th June 2015.

### Outcome and data record-linkage

We performed a data record linkage between the Center database and the health database of the Local Health Authority (LHA) of Brescia based on patient’s social security number in order to compare occurrence of wheezing-related events before and after the DTEP intervention. Outcomes included: a) hospitalizations, with primary or secondary discharge diagnosis of dyspnea or respiratory symptoms; b) outpatient services, spirometry when performed, skin prick testing, total and specific IgE (i.e. ImmunoCAP or microarray ISAC); c) emergency room visits, with primary or secondary diagnosis of dyspnea or respiratory symptoms; and d) drug prescription usage, including inhaled short-acting beta-2 agonists (SABAs), inhaled corticosteroids, long acting beta-2 agonists (LABAs) plus corticosteroids, leukotriene receptor antagonists and systemic corticosteroids. Hospital discharge data were available from 1st September 2007 to 31st December 2014, whereas all the other data were available from 1st January 2010 to 31st December 2014. Onset of the wheezing symptoms was defined as the date of the first wheezing-related event (i.e., hospitalization, use of outpatient services, access to the emergency room and drugs prescription).

### Data analysis

Analyses of data were performed separately in patients aged 0–2 and 3–5 years since management approaches to wheezing symptoms differed by age. Age of children at diagnosis was calculated by the difference between the date of the first wheezing-related event and birthdate. Only children in whom the first occurred in observation period (incident cases) were included in the analysis.

Rates of hospitalizations, emergency room visits, outpatient admissions and drugs consumption were computed as the number of events divided by the person-time throughout the period, multiplied by 1000 (i.e., events per 1000 person-years). Person-years were computed as the sum of the observational events in the period for each subject, from wheezing onset to the end of the observational period (i.e., 31st December 2014). The number of person-years varied for hospitalizations, emergency room visits, outpatient admissions and drugs consumption because of the different dates in which the events occurred. The incidence rates of each outcome before and after the DTEP intervention were computed. The 95% CI values of the incidence rates were calculated assuming a Poisson distribution. The ratios of after-to-before DTEP incidence rates were calculated for each outcome variable (i.e., incidence rate ratio, IRR), together with corresponding 95% CI values, using a Poisson regression for repeated measures. Two-sided exact significance tests were performed for IRRs and all data analyses were performed using the Stata program, version 14.

## Results

A total of 1103 patients aged 0–5 years attended the Center “Io e l’Asma” from 1st September 2007 to 31st December 2014, including 600 patients aged 0–2 years (375 males) and 503 patients (314 males) aged 3–5 years. Among them, 741 patients (67.2%) completed the DTEP, receiving 3 or more evaluations, including 391 and 350 children aged 0–2 and 3–5 years, respectively. Using the hospital discharge data covering the longest observational period, follow-up time was 2837.7 person-years for 0–2 years old and 2772.5 person-years for 3–5 years old subjects, with a mean of 4.7 and 5.5 years for each subject respectively.

The control of wheezing symptoms of children undergoing the DTEP was relatively low at the 1st visit, and increased significantly up to the 3rd visit, as shown in Table 1. The percentage of children with well controlled wheezing symptoms increased from visit 1 to visit 3 in both age groups, from 39.5 to 60.9% and from 25.5 to 75.5% in children aged 0–2 and 3–5 years, respectively.

**Table 1:** Wheezing control of children following the DTEP at 1st and 3rd visit according to age at first visit

Wheezing control	0–2 years old children*		3–5 years old children*	
	1st visit No. (%)	3rd visit No. (%)	1st visit No. (%)	3rd visit No. (%)
Uncontrolled	117 (29.9)	32 (8.3)	93 (26.6)	23 (6.6)
Partly controlled	96 (24.5)	70 (17.8)	102 (29.1)	60 (17)
Well controlled	154 (39.5)	238 (60.9)	89 (25.5)	264 (75.5)
Not assessed	24 (6.1)	51 (13)	66 (18.7)	3 (0.9)
Total	391 (100)	391 (100)	350 (100)	350 (100)

*p < 0.001 by chi square test

Shown in Table 2 are the IRs of wheezing-related outcomes before and after DTEP together with the corresponding IRR in 0–2 and 3–5 years old patients who completed the DTEP for each outcome variable. A statistically significant reduction in the IRs was observed from before to after DTEP for all the outcomes, from − 8.6% to − 80.4%. IRs increased in children aged 0–2 years (+ 22.3%) and did not change in those aged 3–5 years (− 4.7% not statistically significant). Accordingly, the IRRs showed a statistically significant decrease of the risk of wheezing-related outcomes from before to after DTEP for all the outcomes except outpatient services.

**Table 2:** Incidence rate (IR) of wheezing-related outcomes before and after following a Diagnostic Therapeutic Educational Pathway (DTEP), % differences between IRs and incidence rate ratios (IRRs) of after-to-before DTEP IRs, according to children’s age

Health outcome	Before-DTEP		After-DTEP		% difference after-before DTEP rates	IRR (95% CI)^a^	*p*-value
Age (years)	No.	IR per 1000 (95% CI)	No.	IR per 1000 (95% CI)			
Hospitalization							
0–2	198	241.9 (209.4–278.0)	71	65.5 (51.2–82.7)	−72.9	0.27 (0.20–0.36)	*p* < 0.0001
3–5	101	162.9 (132.7–197.9)	43	31.9(23.1–43.0)	−80.4	0.20 (0.13–0.28)	*p* < 0.0001
Emergency room visit							
0–2	389	564.4 (509.7–623.4)	261	256.1 (226.0–289.2)	−54.6	0.45 (0.39–0.53)	p < 0.0001
3–5	99	202.9 (164.9–247.1)	145	121.8 (102.7–143.3)	−40.0	0.60 (0.46–0.78)	*p* = 0.0001
Outpatient services							
0–2	293	425.1 (377.8–476.7)	530	520.1 (476.8–566.3)	+ 22.3	1.22 (1.06–1.42)	*p* = 0.0053
3–5	172	352.6 (301.9–409.4)	400	335.9 (303.8–370.5)	−4.7	0.95 (0.79–1.15)	*p* = 0.59
Drug Prescription							
0–2	10,532	13,156.0 (12,905.9–13,409.7)	12,784	12,025.4 (11,817.8–12,235.7)	−8.6	0.91 (0.89–0.94)	p < 0.0001
3–5	6670	11,175.4 (10,910.6–11,445.0)	10,264	8223.2 (8067.5–8381.0)	−26.4	0.74 (0.71–0.76)	p < 0.0001

*DTEP* Diagnostic-Therapeutic-Educational Pathway, *CI* Confidence interval. ^a^Poisson regression for repeated measures

The rates of drug prescriptions before and after following the DTEP are shown in Table 3. These rates decreased significantly from before to after DTEP for LABA plus corticosteroids and antibiotics in both 0–2 and 3–5 age groups. In contrast, an increase in the use of inhaled short-acting beta-2 agonist (+ 11.3%), inhaled corticosteroids (+ 6.7%) and leukotriene receptor antagonist (+ 103.5%) was observed among children aged 0–2 years from before to after DTEP but not in those aged 3–5 years. The systemic steroid prescription rate decreased in children aged 3–5 years (− 37.4%) but not in children aged 0–2 years (− 4%).

**Table 3:** Incidence rates (IRs) of drug prescription before and after following a Diagnostic Therapeutic Educational Pathway (DTEP), % differences between IRs and incidence rate ratios (IRRs) of after-to-before DTEP IRs, according to children’s age

Drug	Age (years)		Before DTEP		After DTEP			
		No.	Rate per 1000 (95% CI)	No.	Rate per 1000 (95% CI)	% difference after-before DTEP rates	IRR (95% CI)^a^	*p*-value
Inhaled short-acting beta-2 agonist (SABA)								
	0–2	1459	1822.5 (1730.2–1918.5)	2157	2029.0 (1944.3–2116.5)	+ 11.3	1.11 (1.04–1.19)	*p* = 0.0015
	3–5	1061	1754.0 (1650.0–1862.8)	2259	1748.5 (1677.1–1822.1)	−0.3	1.00 (0.93–1.07)	NS
Inhaled corticosteroids								
	0–2	1946	2430.8 (2324.0–2541.3)	2757	2593.4 (2497.5–2692.0)	+ 6.7	1.07 (1.01–1.06)	*p* = 0.0286
	3–5	1284	2122.7 (2008.1–2242.0)	2734	2116.2 (2037.6–2197.0)	−0.3	1.00 (0.93–1.07)	NS
LABA plus corticosteroids								
	0–2	242	302.3 (265.4–342.9)	244	229.5 (201.6–260.2)	−24.1	0.76 (0.63–0.91)	*p* = 0.0025
	3–5	213	352.1 (306.4–402.7)	325	251.6 (225.0–280.5)	−28.6	0.71 (0.60–0.85)	*p* = 0.0002
Leukotriene receptor antagonists								
	0–2	600	749.5 (690.7–811.9)	1621	1524.8 (1451.5–1600.9)	+ 103.5	2.03 (1.85–2.24)	*p* < 0.0001
	3–5	497	821.6 (751.0–897.1)	991	765.1 (720.0–816.3)	−6.6	0.93 (0.84–1.04)	NS
Systemic steroids								
	0–2	710	886.9 (822.9–954.6)	905	851.3 (796.7–908.6)	−4.0	0.96 (0.87–1.06)	NS
	3–5	443	732.3 (665.7–803.8)	592	458.2 (422.1–496.7)	−37.4	0.63 (0.55–0.71)	*p* < 0.0001
Antibiotics								
	0–2	5575	6964.0 (6782.4–7149.2)	5100	4797.4 (4666.6–4930.9)	−31.1	0.69 (0.66–0.72)	*p* < 0.0001
	3–5	3262	5392.6 (5209.1–5580.9)	3723	2881.7 (2789.4–2975.8)	−46.6	0.53 (0.51–0.56)	*p* < 0.0001

*DTEP* Diagnostic-Therapeutic-Educational Pathway, *LABA* Long-acting beta 2 agonist, *CI* Confidence interval. ^a^Poisson regression for repeated measures, *NS* Not significant (*p* > 0.05)

The total costs of drug prescriptions for treatment of wheezing symptoms per patient per year increased in children aged 0–2 years, from 187.09 to 203.99 euro, and decreased in children aged 3–5 years, from 196.40 to 147.63 euro from before to after DTEP (data not shown in Table).

## Discussion

The results of this real-world evidence study showed a positive impact of a Diagnostic Therapeutic Educational Pathway (DTEP) in preschool children on both control of wheezing symptoms as well as on the incidence of health outcomes, particularly hospitalization and emergency room visits. Several previous randomized clinical trials have shown not only that asthma control can be substantially improved by self-management training, a core component of which is patient education, but also that educational and behavioral interventions can achieve positive results in asthma control in children and adults [6, 16]. We have previously documented a favorable impact of our DTEP on asthma control and asthma-related outcomes among children and adolescents aged 6–17 years [11–15]. Since no previous study had evaluated the impact of a Diagnostic Therapeutic Educational Pathway (DTEP) on asthma control among children of less than 6 years so far, the present study, to our knowledge, is the first to do so.

The before-DTEP IRs were particularly elevated especially among the youngest children with IRs for emergency room visit higher than 500/1000 person-years in children aged 0–2 years. This means that about one of two patients had required emergency room visits each year prior to the DTEP intervention. Moreover, the before-DTEP IRs for the other outcomes were higher among children aged 0–2 years than those aged 3–5 years, and much higher than those aged more than 6 years, as reported in another study [14]. These findings agree with previous observations which showed a more frequent use of health services and consumption of health-care resources in preschool than in older children and adults [7–9].

The comparison of the before- and after-DTEP frequencies of health outcomes showed that the proportion of children with well-controlled wheezing symptoms approximately doubled from the 1st to 3rd visit, from 39.5 to 60.9% and from 25.5 to 75.5% in children aged 0–2 and 3–5 years, respectively. Accordingly, the IRs of hospitalization and emergency room visits were halved from before- to after-DTEP, especially in the youngest population, and the use of drugs, not proper to treat wheezing, as LABA plus corticosteroids and antibiotics, declined, especially among 3–5 years old children.

The optimal pharmacologic treatment regimen for preschool children with wheezing continues to remain unclear. The GINA guidelines for asthma management devote a specific chapter to diagnosis and treatment of asthma in children aged 5 years and younger [1]. A recent multicenter, randomized, clinical trial on children aged 12 to 59 months with clinically diagnosed asthma, due to their caregivers’ reporting of daytime symptoms, nighttime awakening or wheezing episodes, necessitating treatment with daily controller therapy, showed that individualized therapy based on determination of aeroallergen sensitization and blood eosinophil may be more beneficial than uniform treatment [17].

In our Center, young children with wheezing are prescribed pharmacologic treatment according to the GINA recommendations and symptom control levels; drug prescription changed from before, during and after DTEP accordingly, though some differences were noted between 0 and 2 and 3–5 years old ones. A statistically significant reduction of drug prescriptions, e.g. LABA plus corticosteroids, antibiotics and systemic steroids, that are often over-prescribed, emerged in both age groups.

Important differences were observed according to category of drugs. The prescription of drugs for management of wheezing attack, such as inhaled short-acting beta-2 agonist (SABA), increased in 0–2 years old children, from before to after-DTEP, suggesting a more frequent recognition of symptoms in them, whereas it did not vary in those aged 3–5 years, probably because the wheezing symptoms had been already recognized by their primary care physicians in the period before DTEP. The rate of systemic corticosteroid usage, commonly prescribed for management of the wheezing attack, decreased in both groups, especially in 3–5 years old children, probably due to a clearer clinical pattern and the choice of SABA as the first line treatment.

Prescription of inhaled corticosteroids increased in the 0–2 age group, from before to after DTEP, possibly for control of wheezing, whereas it did not vary in the 3–5 age group most likely because the symptoms remained well controlled without daily therapy due to an appropriate wheezing management, and with the interaction between specialists and primary care physicians [1, 3, 13, 18]. The increase of daily therapy with leukotriene receptor antagonists in 0–2 years old subjects is consistent with GINA recommendations, since these medications are considered as other controller options for obtaining and maintaining wheezing control associated with upper respiratory tract infections (i.e. laryngitis or rhinitis). The rate did not vary significantly in the 3–5 age group possibly due to a better control of symptoms without daily drug therapy.

The control of wheezing symptoms achieved and maintained with first-line therapy, specifically inhaled corticosteroid at lower dosage or leukotriene receptor antagonist, was followed by a decrease in the prescriptions of daily therapy with LABA plus corticosteroids, in both groups. This finding is consistent with GINA recommendations that there are insufficient data about the efficacy and safety of combination ICS/long- acting beta2-agonist (LABA) in this age group to recommend their use. Antibiotic prescriptions, which were largely inconsistent with GINA guidelines, were reduced following improved symptom control.

Although the results of this clinical and implementation research study showed some positive impact of the DTEP, it had some limitations. The retrospective design and the absence of a control group limit generalizations that can be drawn from these results. At the same time, the current and complete documentation of clinical data at each visit, using a dedicated software and the record linkage with the LHA database for computing the rates of wheezing-related health outcomes, reduced risk of information bias. Future randomized controlled studies will be required to more definitively delineate the effectiveness of a DPTE pathway intervention in the management of wheezing in children aged less than 6 years.

## Conclusions

This real-world study suggests that in children aged 5 years or less, an integrated diagnostic, therapeutic and educational pathway shared with primary care physicians improves wheezing control and reduces the frequency of asthma-related adverse outcomes.

## Data Availability

Not applicable.
